# ID 98 - População com Acesso a Medicamentos Especializados no Sistema Único de Saúde (SUS): um estudo epidemiológico nacional

**DOI:** 10.5327/2237-9622.2025.v34s1.98

**Published:** 2025-11-25

**Authors:** Ivan Ricardo Zimmermann, Rosângela Maria Gomes, Stéfani Sousa Borges, Patricia Silveira Rodrigues, Karen Sarmento Costa, Noemia Urruth Leão Tavares

**Keywords:** acesso a medicamentos, componente especializado, alto custo, equidade em saúde, Sistema Único de Saúde

## Abstract

**Introdução:**

O Componente Especializado da Assistência Farmacêutica (Ceaf) é uma das políticas nacionais de acesso a medicamentos no SUS, que disponibiliza medicamentos de forma equitativa à população brasileira, independentemente de seu custo unitário. Como política pública, busca-se o equilíbrio entre a sustentabilidade financeira do sistema de saúde e o direito dos cidadãos aos tratamentos necessários de forma integral, especialmente para doenças raras, crônicas ou de alta complexidade. O presente estudo busca descrever as características demográficas e epidemiológicas da população atendida pelo Ceaf no ano de 2023.

**Método:**

Foi conduzido um estudo observacional transversal de base populacional com dados do Departamento de Informática do Sistema Único de Saúde (DataSUS). Para tanto, foram acessados os microdados dos registros de autorização de procedimentos ambulatoriais (Apac) processados durante todo o ano de 2023. Com auxílio do pacote read.dbc e da linguagem R, todos os registros disponíveis foram extraídos e individualizados por códigos de Cartão Nacional de Saúde (CNS) e suas respectivas características demográficas (UF, sexo, idade e raça/cor) e epidemiológicas de acordo com a Classificação Internacional de Doenças e Problemas Relacionados à Saúde (CID-10). Todos os dados acessados se encontram disponíveis publicamente de forma anônima e desidentificada, respeitando os princípios éticos e de proteção de dados.

**Resultados:**

O Ceaf processou a dispensação de medicamentos especializados para um total de 3.336.776 pacientes no Brasil. O número de pacientes atendidos por cada unidade da Federação (UF) variou de 0,1% (2.943) no estado do Amapá a 41,8% (1.394.006) no estado de São Paulo. A idade média da população total atendida foi de 54,3 ± 20,3, variando em sua média de 40,6 ± 19,6 em Tocantins a 57,3 ± 22,6 em São Paulo. A maioria da população foi identificada pelo sexo feminino (58,7%). Em relação à raça/cor, 58,7% dos pacientes autodeclararam-se como branca (58,0%), 34,5% parda, 5,0% preta, 2,5% amarela e 0,03% indígena. Em 7,9% da população do estudo não foi encontrado a informação da raça/cor. As condições clínicas mais atendidas foram hipercolesterolemia pura (7,7%), esquizofrenia paranoide (7,4%), asma predominantemente alérgica (6,7%), glaucoma primário de ângulo aberto (5,4%) e doença renal em estádio final (5,3%).

**Conclusão:**

O estudo destaca as grandes dimensões do Ceaf, atendendo uma população de mais de 3 milhões de pessoas durante o ano de 2023. Foram observadas algumas diferenças regionais relevantes na demanda, como o grande contraste entre o estado de São Paulo, que atendeu 41,8% do total de pacientes, e o Amapá, com apenas 0,1%. A média de idade de 54,3 anos dos pacientes sugere uma predominância de doenças crônicas que afetam principalmente a população mais velha, enquanto a maior presença de mulheres (58,7%) pode refletir tanto a longevidade feminina quanto possíveis barreiras de acesso enfrentadas pelo sexo masculino. A análise demonstra também um predomínio de pacientes que se autodeclaram brancos e pardos, refletindo a demografia nacional. As condições clínicas atendidas, como hipercolesterolemia pura e esquizofrenia paranoide, destacam a particularidade de um sistema que atende de forma integral uma variedade de necessidades de saúde complexas. Tais resultados ratificam a importância do Ceaf como política pública de acesso a medicamentos em busca da equidade da saúde no Brasil.

